# Supplementary material for: POLD1 DEDD Motif Mutation Confers Hypermutation in Endometrial Cancer and Durable Response to Pembrolizumab
Source: Cancers (Basel). 2023 Nov 30;15(23):5674. doi: 10.3390/cancers15235674 (PMC10705788; doi:10.3390/cancers15235674)
Supplement: Supplementary file 1 [file cancers-15-05674-s001.zip › cancers-2736324-supplementary.pptx]

## Slide 1
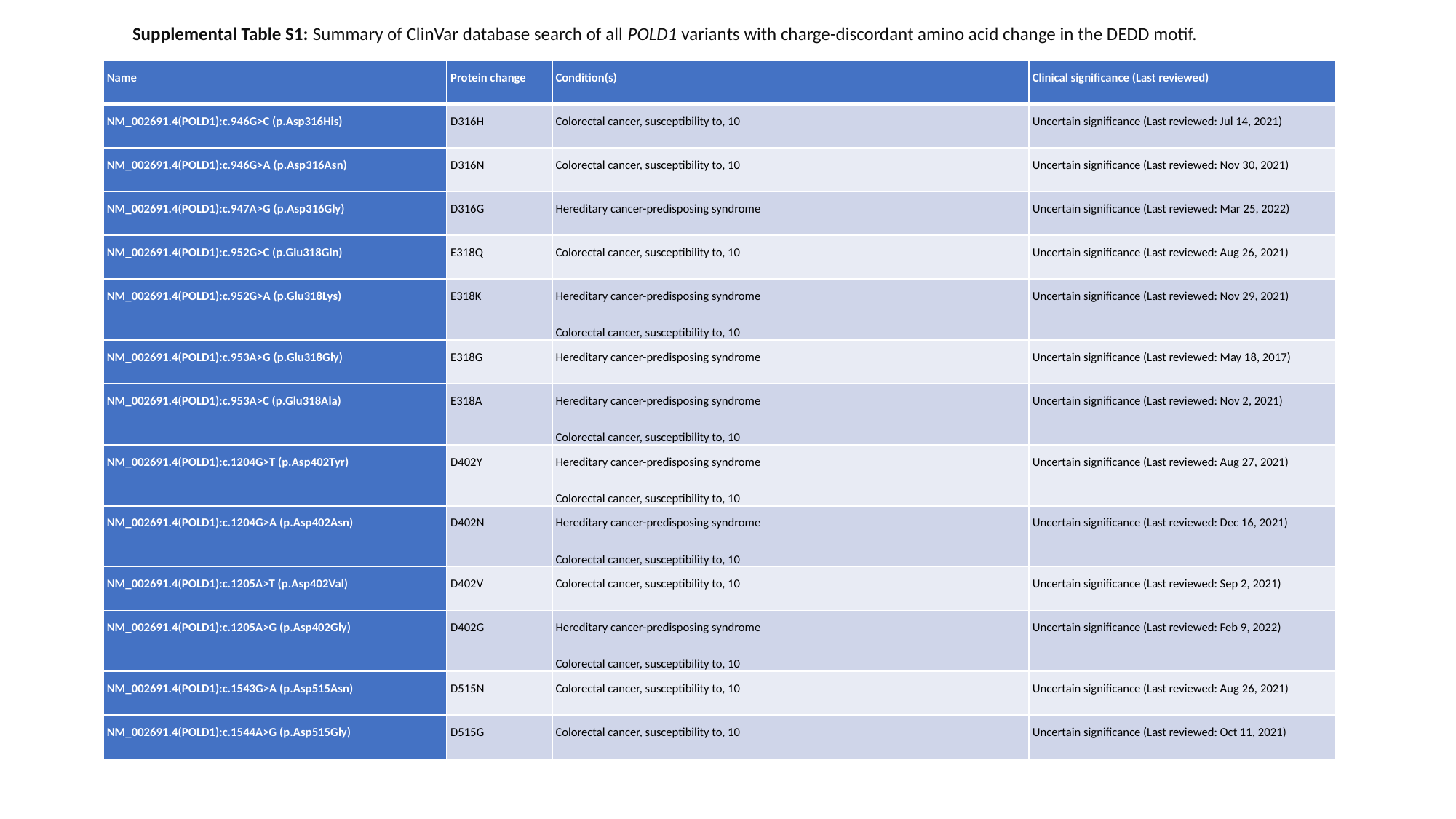

Supplemental Table S1: Summary of ClinVar database search of all POLD1 variants with charge-discordant amino acid change in the DEDD motif.
| Name | Protein change | Condition(s) | Clinical significance (Last reviewed) |
| --- | --- | --- | --- |
| NM\_002691.4(POLD1):c.946G>C (p.Asp316His) | D316H | Colorectal cancer, susceptibility to, 10 | Uncertain significance (Last reviewed: Jul 14, 2021) |
| NM\_002691.4(POLD1):c.946G>A (p.Asp316Asn) | D316N | Colorectal cancer, susceptibility to, 10 | Uncertain significance (Last reviewed: Nov 30, 2021) |
| NM\_002691.4(POLD1):c.947A>G (p.Asp316Gly) | D316G | Hereditary cancer-predisposing syndrome | Uncertain significance (Last reviewed: Mar 25, 2022) |
| NM\_002691.4(POLD1):c.952G>C (p.Glu318Gln) | E318Q | Colorectal cancer, susceptibility to, 10 | Uncertain significance (Last reviewed: Aug 26, 2021) |
| NM\_002691.4(POLD1):c.952G>A (p.Glu318Lys) | E318K | Hereditary cancer-predisposing syndrome Colorectal cancer, susceptibility to, 10 | Uncertain significance (Last reviewed: Nov 29, 2021) |
| NM\_002691.4(POLD1):c.953A>G (p.Glu318Gly) | E318G | Hereditary cancer-predisposing syndrome | Uncertain significance (Last reviewed: May 18, 2017) |
| NM\_002691.4(POLD1):c.953A>C (p.Glu318Ala) | E318A | Hereditary cancer-predisposing syndrome Colorectal cancer, susceptibility to, 10 | Uncertain significance (Last reviewed: Nov 2, 2021) |
| NM\_002691.4(POLD1):c.1204G>T (p.Asp402Tyr) | D402Y | Hereditary cancer-predisposing syndrome Colorectal cancer, susceptibility to, 10 | Uncertain significance (Last reviewed: Aug 27, 2021) |
| NM\_002691.4(POLD1):c.1204G>A (p.Asp402Asn) | D402N | Hereditary cancer-predisposing syndrome Colorectal cancer, susceptibility to, 10 | Uncertain significance (Last reviewed: Dec 16, 2021) |
| NM\_002691.4(POLD1):c.1205A>T (p.Asp402Val) | D402V | Colorectal cancer, susceptibility to, 10 | Uncertain significance (Last reviewed: Sep 2, 2021) |
| NM\_002691.4(POLD1):c.1205A>G (p.Asp402Gly) | D402G | Hereditary cancer-predisposing syndrome Colorectal cancer, susceptibility to, 10 | Uncertain significance (Last reviewed: Feb 9, 2022) |
| NM\_002691.4(POLD1):c.1543G>A (p.Asp515Asn) | D515N | Colorectal cancer, susceptibility to, 10 | Uncertain significance (Last reviewed: Aug 26, 2021) |
| NM\_002691.4(POLD1):c.1544A>G (p.Asp515Gly) | D515G | Colorectal cancer, susceptibility to, 10 | Uncertain significance (Last reviewed: Oct 11, 2021) |

## Slide 2
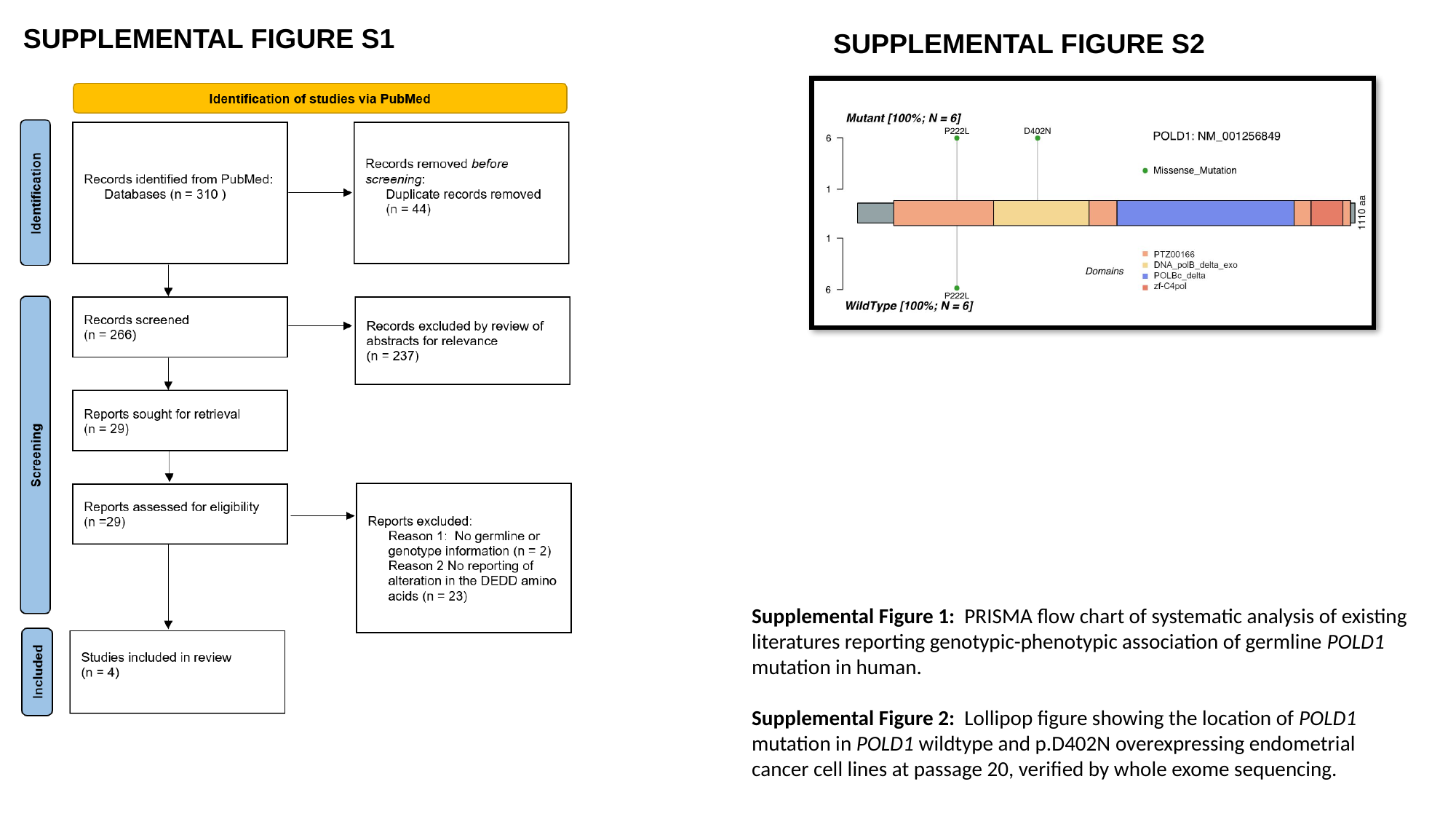

SUPPLEMENTAL FIGURE S1
SUPPLEMENTAL FIGURE S2
Supplemental Figure 1: PRISMA flow chart of systematic analysis of existing literatures reporting genotypic-phenotypic association of germline POLD1 mutation in human.
Supplemental Figure 2: Lollipop figure showing the location of POLD1 mutation in POLD1 wildtype and p.D402N overexpressing endometrial cancer cell lines at passage 20, verified by whole exome sequencing.
