# Supplementary material for: POLD1 DEDD Motif Mutation Confers Hypermutation in Endometrial Cancer and Durable Response to Pembrolizumab
Source: Cancers (Basel). 2023 Nov 30;15(23):5674. doi: 10.3390/cancers15235674 (PMC10705788; doi:10.3390/cancers15235674)
Supplement: Supplementary file 1 [file cancers-15-05674-s001.zip › Supplemental Figure S3.pdf]

```
P28340.2  1    M    DGKRRPGPGPGVPPKRARGGlwDDDDAPRPSQFEEDLALMEEME-AEHRLQEQEEEELQSVLE[12]DPRWLRPT  83
P52431.2  1    M    DCKRRQGPGPGVPPKRARGHlwDEDE-PSPSQFEANLALLEEIE-AENRLQEAEEE-LQLPPE[12]DPRWRRPT  81
P54358.2  1    M    DGKRKFNGTSNGHAKKPRNP--DDDE---EMGFEAELAAFENSEdMDQTLLMGDGPENQTTSE    --RWSRPP  65
P90829.1  1    M[ 1]-SKRPGGSSFQPEVKRKRES--DEFEQCYVSRFENELPSVPTID------------------    KTGWARPA  51
P15436.4  1    M[47]DSFRKYNS----QGFKAKDT--DLMGTQLESTFEQELSQMEHDM------ADQEEHDLSSFER    -----KKL  102

P28340.2  84   PPALDPQTEPLIFQQLEIDHYVGPaQPVPGGPPPSRGSVPVLRAFGVTDEGFSVCCHIHGFAPYFYTPAPPGFGPEHMGD  163
P52431.2  82   LRALDPSTEPLIFQQLEIDHYVGSaPPLPEGPLPSRNSVPILRAFGVTDEGFSVCCHIQGFAPYFYTPAPPGFGAEHLSE  161
P54358.2  66   PPELDPSKHNLEFQQLDVENYLG--QPLPGMPGAQIGPVPVVRMFGVTMEGNSVCCHVHGFCPYFYIEAPSQFEEHHCEK  143
P90829.1  52   VDKDLGISKSIACQILEVETYHED-------GSATSYDRTNVKLYGVTKSGNSICVIVTDYFPHFYFQAPQGFGVEHIGT  124
P15436.4  103  PTDFDPSLYDISFQQIDAEQSVLN-------GIKDENTSTVVRFFGVTSEGHSVLCNVTGFKNYLYVPAPNSSDANDQEQ  175

P28340.2  164  LQREL-NLAISRDSRGGR---ELTGPAV---LAVELCSRESMFGYHG-HGPSPFLRITVALPRLVAPARRLLEQGIRVAG  235
P52431.2  162  LQQEL-NAAISRDQRGGK---ELSGPAV---LAIELCSRESMFGYHG-HGPSPFLRITLALPRLMAPARRLLEQGVRVPG  233
P54358.2  144  LQKAL-DQKVIADIRNNK---DNVQEAV---LMVELVEKLNIHGYNG-DKKQRYIKISVTLPRFVAAASRLLKKEVIMSE  215
P90829.1  125  AQSAIcNMVAAAKRRGGSgqaQLPGKVVdnlVHVEIVHGENLYYFRGaDTKVPFVKVSGSTEAL-HKARMELKNGVNLMG  203
P15436.4  176  INKFV------------HylnETFDHAId---SIEVVSKQSIWGYSG-DTKLPFWKIYVTYPHMVNKLRTAFERGHLSFN  239

P28340.2  236  LGTPSFAP-YEANVDFEIRFMVDTDIVGCNWLELPAGKYALRLKEKA----TQCQLEADVLWSDVVSHPPEGPWQRIAPL  310
P52431.2  234  LGTPSFAP-YEANVDFEIRFMVDADIVGCNWLELPAGKYVRRAEKKA----TLCQLEVDVLWSDVISHPPEGQWQRIAPL  308
P54358.2  216  IDFQDCRA-FENNIDFDIRFMVDTDVVGCNWIELPMGHWRIRNSHSKPlpeSRCQIEVDVAFDRFISHEPEGEWSKVAPF  294
P90829.1  204  KGPVNVGNlYESNINVIVMFLAKTNIVGCGWIEIPAGKCRILSNSEKS---SRCQIEVTVPVKNLIVHESDGEWAGIAPI  280
P15436.4  240  SWFSNGTTtYD-NIAYTLRLMVDCGIVGMSWITLPKGKYSMIEPNNRV---SSCQLEVSINYRNLIAHPAEGDWSHTAPL  315

P28340.2  311  RVLSFDIECAGRKGIFPEPERDPVIQICSLGLRWGEPEPFLRLALTLRPCAPILGAKVQSYEKEEDLLQAWSTFIRIMDP  390
P52431.2  309  RVLSFDIECAGRKGIFPEPERDPVIQICSLGLRWGEPEPFLRLALTLRPCAPILGAKVQSYEREEDLLQAWADFILAMDP  388
P54358.2  295  RILSFDIECAGRKGIFPEAKIDPVIQIANMVIRQGEREPFIRNVFTLNECAPIIGSQVLCHDKETQMLDKWSAFVREVDP  374
P90829.1  281  RTLSLDIECIGRRGVFPEAIKDPIIQIANLVKIEGEAEPFVRNCFVLGTCAPVVGSNIIQCVNEKVLLEKWAEFVREVDP  360
P15436.4  316  RIMSFDIECAGRIGVFPEPEYDPVIQIANVVSIAGAKKPFIRNVFTLNTCSPITGSMIFSHATEEEMLSNWRNFIIKVDP  395

P28340.2  391  DVITGYNIQNFDLPYLISRAQTLKVQTFPFLGRVAGLCSNIRDSSFQSKQTGRRDTKVVSMVGRVQMDMLQVLLREYKLR  470
P52431.2  389  DVITGYNIQNFDLPYLISRAQALKVDRFPFLGRVTGLRSNIRDSSFQSRQVGRRDSKVISMVGRVQMDMLQVLLREHKLR  468
P54358.2  375  DILTGYNINNFDFPYLLNRAAHLKVRNFEYLGRIKNIRSVIKEQMLQSKQMGRRENQYVNFEGRVPFDLLFVLLRDYKLR  454
P90829.1  361  DIITGYNILNFDLPYILDRAKVLSLPQVSHLGRQKEKGSVVRDAAISSKQMGSRVNKSIDIHGRIIFDVLQVVLRDYKLR  440
P15436.4  396  DVIIGYNTTNFDIPYLLNRAKALKVNDFPYFGRLKTVKQEIKESVFSSKAYGTRETKNVNIDGRLQLDLLQFIQREYKLR  475

P28340.2  471  SYTLNAVSFHFLGEQKEDVQHSIITDLQNGNDQTRRRLAVYCLKDAYLPLRLLERLMVLVNAVEMARVTGVPLSYLLSRG  550
P52431.2  469  SYTLNAVSFHFLGEQKEDVQHSIITDLQNGNEQTRRRLAVYCLKDAFLPLRLLERLMVLVNNVEMARVTGVPLGYLLTRG  548
P54358.2  455  SYTLNAVSYHFLQEQKEDVHHSIITDLQNGDEQTRRRLAMYCLKDAYLPLRLLEKLMAIVNYMEMARVTGVPLESLLTRG  534
P90829.1  441  SYTLNSVSYQFLSEQKEDVEHNIIPDLQRGDEQTRRRLAQYCLKDAYLPLRLLDKLMSIINYIEMARVTGVPMNFLLTKG  520
P15436.4  476  SYTLNAVSAHFLGEQKEDVHYSIISDLQNGDSETRRRLAVYCLKDAYLPLRLMEKLMALVNYTEMARVTGVPFSYLLARG  555

P28340.2  551  QQVKVVSQLLRQAMHEGLLMPVVKSEGG--EDYTGATVIEPLKGYYDVPIATLDFSSLYPSIMMAHNLCYTTLLRPGTAQ  628
P52431.2  549  QQVKVVSQLLRQAMRQGLLMPVVKTEGS--EDYTGATVIEPLKGYYDVPIATLDFSSLYPSIMMAHNLCYTTLLRPGAAQ  626
P54358.2  535  QQIKVLSQLLRKAKTKGFIMPSYTSQGSD-EQYEGATVIEPKRGYYADPISTLDFASLYPSIMMAHNLCYTTLVLGGTRE  613
P90829.1  521  QQIKILSMMLRRCKQNNFFLPVIEANSGDgEGYEGATVIDPIRGFYNEPIATLDFASLYPSIMIAHNLCYTTLLKSPQ--  598
P15436.4  556  QQIKVVSQLFRKCLEIDTVIPNMQSQASD-DQYEGATVIEPIRGYYDVPIATLDFNSLYPSIMMAHNLCYTTLCNKATVE  634

P28340.2  629  KL   GLTEDQFIRTPTGDEFVKTSVRKGLLPQILENLLSARKRAKAELAKETDPLRRQVLDGRQLALKVSANSVYGFTG  705
P52431.2  627  KL   GLKPDEFIKTPTGDEFVKSSVRKGLLPQILENLLSARKRAKAELAQETDPLRRQVLDGRQLALKVSANSVYGFTG  703
P54358.2  614  KL[4]NLQDDQVERTPANNYFVKSEVRRGLLPEILESLLAARKRAKNDLKVETDPFKRKVLDGRQLALKISANSVYGFTG  694
P90829.1  599  --   GVENEDYIRTPSGQYFATKSKRRGLLPEILEDILAARKRAKNDMKNEKDEFKRMVYNGRQLALKISANSVYGFTG  673
P15436.4  635  RL[3]--IDEDYVITPNGDYFVTTKRRRGILPIILDELISARKRAKKDLRDEKDPFKRDVLNGRQLALKISANSVYGFTG  712

P28340.2  706  AQVGKLPCLEISQSVTGFGRQMIEKTKQLVESKYT---VENGYSTSAKVVYGDTDSVMCRFGVSSVAEAMALGREAADWV  782
P52431.2  704  AQVGKLPCLEISQSVTGFGRQMIEKTKQLVESKYT---VENGYDANAKVVYGDTDSVMCRFGVSSVAEAMSLGREAANWV  780
P54358.2  695  AQVGKLPCLEISGSVTAYGRTMIEMTKNEVESHYT---QANGYENNAVVIYGDTDSVMVNFGVKTLERSMELGREAAELV  771
P90829.1  674  ATVGKLPCLEISQSVTAFGRKMIDMTKLEVERIYKkgaLDGKCPADAKVIYGDTDSVMVKFGVETVAQAMEIGLDAAKEV  753
P15436.4  713  ATVGKLPCLAISSSVTAYGRTMILKTKTAVQEKYC---IKNGYKHDAVVVYGDTDSVMVKFGTTDLKEAMDLGTEAAKYV  789

P28340.2  783  SGHFPSPIRLEFEKVYFPYLLISKKRYAGLLFSsRPDAHDRMDCKGLEAVRRDNCPLVANLVTASLRRLLIDRDPEGAVA  862
P52431.2  781  SSHFPSPIRLEFEKVYFPYLLISKKRYAGLLFSsRSDAHDKMDCKGLEAVRRDNCPLVANLVTSSLRRILVDRDPDGAVA  860
P54358.2  772  SSKFVHPIKLEFEKVYYPYLLINKKRYAGLYFT-RPDTYDKMDCKGIETVRRDNSPLVANLMNSCLQKLLIERDPDGAVA  850
P90829.1  754  SKIFTPPIKLEFEKVYSPYLLINKKRYAGLYFT-KPDVHDKMDCKGLETVRRDNCPLVAKVLGVCLEKLLIERDQQSALD  832
P15436.4  790  STLFKHPINLEFEKAYFPYLLINKKRYAGLFWT-NPDKFDKLDQKGLASVRRDSCSLVSIVMNKVLKKILIERNVDGALA  868

P28340.2  863  HAQDVISDLLCNRIDISQLVITKELTRAASDYAGKQAHVELAERMRKRDPGSAPSLGDRVPYVIISAAKGVAAYMKSEDP  942
P52431.2  861  HAKDVISDLLCNRIDISQLVITKELTRAAADYAGKQAHVELAERMRKRDPGSAPSLGDRVPYVIIGAAKGVAAYMKSEDP  940
P54358.2  851  YVKQVIADLLCNRIDISHLVITKELAK--TDYAAKQAHVELAAKMKKRDPGTAPKLGDRVPYVICAAAKNTPAYQKAEDP  928
P90829.1  833  FAKRTISDLLCNKIDISLLIISKELTKSGDKYQAKQAHVELAARMKKRDAGSAPRLGDRVPYVFVAAAKNVPAYERAEDP  912
P15436.4  869  FVRETINDILHNRVDISKLIISKTLAP---NYTNPQPHAVLAERMKRRE-GVGPNVGDRVDYVIIGG--NDKLYNRAEDP  942
```

P28340.2	943	LFVLEHSLPIDTQYYLEQQLAKPLLRIFEPILGEGRAEAVLLRGDHTRCKTVLTGKVGGLLAFAKRRNCCIGCRTVLS--	1020
P52431.2	941	LFVLEHSLPIDTQYYLEQQLAKPLLRIFEPILGEGRAESVLLRGDHTRCKTVLTSKVGGLLAFTKRRNCCIGCRSVID--	1018
P54358.2	929	LYVLENSVPIDATYYLEQQLSKPLLRIFEPILGDN-AESILLKGEHTRTRTVVTSKVGGLAGFMTKKTSCLGCKSLMPKG	1007
P90829.1	913	TFVLQNNIPLDTKHYLTNQLAKPLARIFEPILGD-RAEKILVEGEHTRVRTVVQSKVGGLAAFTTKSATCLGCKSVLPRA	991
P15436.4	943	LFVLENNIQVDSRYYLTNQLQNPIISIVAPIIGDKQANGMFVVKS---IKINTGSQKGGLMSFIKKVEACKSCKGPLRKG	1019
P28340.2	1021	-HQGAVCEFCQPRESELYQKEVSHLNALEERFSRLWTQCQRCQGSLHEDVICTSRDCPIFYMRKKVRKDLEDQEQLLRRF	1099
P52431.2	1019	-HQGAVCKFCQPRESELYQKEVSHLNALEERFSRLWTQCQRCQGSLHEDVICTSRDCPIFYMRKKVRKDLEDQERLLQRF	1097
P54358.2	1008	yEQACLCPHCEPRMSELYQKEVGAKRELEETFSRLWTECQRCQESLHEEVICSNRDCPIFYMRQKVRMDLDNQEKRVLRF	1087
P90829.1	992	eSENAVCKHCEPKLPTIFASRMNTMHELENHFGRLWTECQNCAKTMQDKVNCSARDCPIYYMREKVRNELSEASAVIERF	1071
P15436.4	1020	--EGPLCSNCLARSGELYIKALYDVRDLEEKYSRLWTQCQRCAGNLHSEVLCSNKNCDIFYMRVKVKKELQEKVEQLSKW	1097
P28340.2	1100	GPPGPEAW--	1107
P52431.2	1098	GPPGPEAW--	1105
P54358.2	1088	GLA---EW--	1092
P90829.1	1072	GDPCFQAPtk	1081
P15436.4		----------	

*P28340: Human, P52431: Mouse, P54358: *Drosophila,* P90829: *c. elegans*, P15436: *s. cerevisiae*
