# Supplementary material for: POLD1 DEDD Motif Mutation Confers Hypermutation in Endometrial Cancer and Durable Response to Pembrolizumab
Source: Cancers (Basel). 2023 Nov 30;15(23):5674. doi: 10.3390/cancers15235674 (PMC10705788; doi:10.3390/cancers15235674)
Supplement: Supplementary file 1 [file cancers-15-05674-s001.zip › Supplementary Table S2.pdf]

Supplementary Table S2

***In silico* Prediction Program**	**Website**
PolyPhen-2	http://genetics.bwh.harvard.edu/pph2/
Mutation Taster	https://www.mutationtaster.org/
Panther	https://www.pantherdb.org/
Mutation Assessor	http://mutationassessor.org/r3/
SIFT	https://sift.bii.a-star.edu.sg/www/Extended_SIFT_chr_coords_submit.html
MutPred2 Score	http://mutpred.mutdb.org/#qform
